# Mito-Mendelian interactions alter in vivo glucose metabolism and insulin sensitivity in healthy mice

**DOI:** 10.1152/ajpendo.00069.2021

**Published:** 2021-08-09

**Authors:** Melissa J. Sammy, Ashley W. Connelly, Jamelle A. Brown, Cassie Holleman, Kirk M. Habegger, Scott W. Ballinger

**Affiliations:** ^1^Division of Molecular and Cellular Pathology, Department of Pathology, University of Alabama at Birmingham, Birmingham, Alabama; ^2^Center for Free Radical Biology, University of Alabama at Birmingham, Birmingham, Alabama; ^3^Division of Endocrinology, Diabetes & Metabolism, Department of Medicine, University of Alabama at Birmingham, Birmingham, Alabama

**Keywords:** adiposity, glucose metabolism, insulin sensitivity, mitochondrial DNA, mitochondrial-nuclear eXchange

## Abstract

The regulation of euglycemia is essential for human health with both chronic hypoglycemia and hyperglycemia having detrimental effects. It is well documented that the incidence of type 2 diabetes increases with age and exhibits racial disparity. Interestingly, mitochondrial DNA (mtDNA) damage also accumulates with age and its sequence varies with geographic maternal origins (maternal race). From these two observations, we hypothesized that mtDNA background may contribute to glucose metabolism and insulin sensitivity. Pronuclear transfer was used to generate mitochondrial-nuclear eXchange (MNX) mice to directly test this hypothesis, by assessing physiologic parameters of glucose metabolism in nuclear isogenic C57BL/6J mice harboring either a C57BL/6J (C57^n^:C57^mt^ wild type—control) or C3H/HeN mtDNA (C57^n^:C3H^mt^—MNX). All mice were fed normal chow diets. MNX mice were significantly leaner, had lower leptin levels, and were more insulin sensitive, with lower modified Homeostatic Model Assessment of Insulin Resistance (mHOMA-IR) values and enhanced insulin action when compared with their control counterparts. Further interrogation of muscle insulin signaling revealed higher phosphorylated Akt/total Akt ratios in MNX animals relative to control, consistent with greater insulin sensitivity. Overall, these results are consistent with the hypothesis that different mtDNA combinations on the same nuclear DNA (nDNA) background can significantly impact glucose metabolism and insulin sensitivity in healthy mice.

**NEW & NOTEWORTHY** Different mitochondrial DNAs on the same nuclear genetic background can significantly impact body composition, glucose metabolism, and insulin sensitivity in healthy mice.

## Graphical abstract

**Figure:**
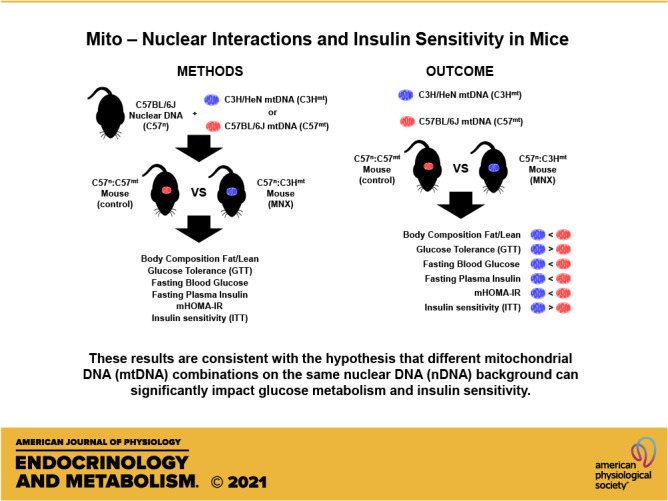

## INTRODUCTION

In the early 1990s, two mitochondrial DNA (mtDNA) mutations were directly linked to diabetes (1, 2) both of which cause maternally inherited diabetes and premature deafness (MIDD), also known as Ballinger–Wallace syndrome (2, 3). These initial reports, although perhaps representing relatively rare forms of diabetes, did demonstrate that mtDNA mutations could impact glucose metabolism. Since then, additional mutations in the mtDNA have been associated with elevated fasting insulin (4), increased fasting plasma glucose (5), increased Homeostatic Model Assessment of Insulin Resistance (HOMA-IR) values (6), as well as type 2 diabetes (7, 8) and metabolic syndrome (6). Racial disparities are also known to exist in terms of susceptibility to diabetes, with non-Hispanic Blacks, Hispanics, and Asians having greater risk for diabetes compared with non-Hispanic Whites (9). Susceptibility to diabetes also increases with age, with the percentage of people with diabetes increasing more than fourfold from 4.2% among people 18–44 yr of age to 17.5% among people 45–64 yr of age and is even higher among the 65 and older group at 26.8% (9). Interestingly, the attributes of both racial disparity and increasing prevalence with age may, in part, be a consequence of the interrelationship of human evolution and mitochondrial functions.

The origins of the eukaryotic cell are the consequence of an estimated 1.5 billion years of endosymbiosis and coevolution between the mitochondrial and nuclear genomes. It has been suggested that this mitochondrial-nuclear interaction or “mito-Mendelian” genetics enabled organismal adaptation to environmental challenges and changes in caloric sources (3, 10, 11). A corollary to this concept is that different mtDNA genetic backgrounds also affect aspects of glucose metabolism. Consistent with these concepts are the findings that mtDNA mutations can be linked with diabetes and/or related risk factors, as discussed in the previous paragraph. Furthermore, it is well established that an individual’s mtDNA background represents their maternal genetic/racial origins (as the mtDNA is maternally inherited). Mitochondrial function declines with age due to sustained oxidative damage over time, along with age-associated decreases in organelle quality control, that collectively contribute to changes in ATP and oxidant production, factors that are both integral for normal glucose metabolism and insulin secretion (12, 13) and have been implicated in type 2 diabetes (14–17).

Recent results using a congenic approach in aged mice supports the notion of different mtDNA backgrounds affecting glucose metabolism (18, 19); however, the impact of different mtDNA backgrounds on glucose metabolism in younger mice has not been examined. Furthermore, congenic approaches do not address the effects introduced by nuclear recombination and allelic selection events that occur during F_1_ hybrid generation, and standard backcrossing strategies, used to produce congenic models. To directly address the impact of different mtDNAs on the same nDNA background on normal glucose metabolism in young mice, we performed pronuclear transfer or “mitochondrial-nuclear eXchange” (MNX) as previously described (20–23) to produce isogenic C57BL/6J mice having different mtDNA backgrounds. This approach is distinct from conplastic (24) or xenomitochondrial (25) approaches in that MNX mice are generated directly with 100% of the desired nuclear and mtDNA complements from respective donor strains through nuclear transfer (20–23). Thus, MNX mice allow for unambiguous assessment of the mtDNA contributions to the pathway of interest since there is neither complexity introduced by potential nuclear crossover nor combinational effects in the filial generations associated with standard backcrossing methods used to generate conplastic mice (20–23).

For these studies, C57BL/6J (C57) control mice were chosen due to their known susceptibility to metabolic diseases (26–30), whereas C3H/HeN mice were chosen as the mtDNA donor as they have been shown to be relatively less susceptible (30–32). C3H mice have been shown to have lower fed plasma insulin and plasma glucose levels and increased glucose tolerance compared with C57BL/6J mice and are considered a diabetes-resistant strain (30–32). Aspects of glucose metabolism were assessed in C57 mice harboring a C57 mtDNA (C57^n^:C57^mt^—control) compared with nuclear isogenic C57 mice harboring a C3H/HeN mtDNA (C57^n^:C3H^mt^—MNX). We hypothesized that C3H mtDNA would confer increased insulin sensitivity in MNX compared with control mice. Consistently, these different mtDNA backgrounds altered aspects of glucose metabolism on the C57BL/6J nDNA background, including exogenous glucose and insulin sensitivity, as well as insulin sensitivity defined by the modified Homeostatic Model Assessment of Insulin Resistance (mHOMA-IR). Changes in body composition and muscle insulin signaling were also consistent with alterations in glucose metabolism, supporting the concept that mtDNA background can significantly influence features of glucose metabolism in vivo.

## MATERIALS AND METHODS

### Mice

All procedures were approved by the University of Alabama at Birmingham Institutional Animal Care and Use Committee. All experiments utilized male mice generated from our mitochondrial-nuclear eXchange (MNX) mouse colony at the University of Alabama at Birmingham. These mice share the same nuclear background (C57^n^) but have either a C57BL6/J or C3H/HeN mtDNA (C57^mt^ or C3H^mt^, respectively), resulting in two nuclear isogenic strains having different mtDNAs (C57^n^:C57^mt^—control, and C57^n^:C3H^mt^—MNX). The generation of the original founders for these colonies has been previously described (20–23), and subsequent generations of MNX and control mice were generated by crossing respective MNX and control females with nuclear matched males so that all animals within an experimental design share the same paternal lineage (e.g., control male siblings were used to sire both the control and MNX progeny to maximize nuclear isogenicity). All offspring are mtDNA haplotyped from genomic DNAs extracted from ear or tail clips to confirm homoplasmy by PCR analysis, as previously described (20–23). For these studies, only male mice were used as female C57 mice are known to be resistant to adverse metabolic phenotypes, including glucose tolerance and insulin resistance (33–35).

### Body Weight, Composition, and Food Consumption

Three-week-old male control and MNX mice were weaned onto a chow diet (Research Diets, Cat. No. D12450J), group housed, and provided food and water ad libitum. Animal weight and food consumption were monitored on a weekly basis from 6 to 18 wk of age during cage changes. In vivo body composition measures were determined by quantitative magnetic resonance (QMR) at 6, 12, and 18 wk of age. For these studies, QMR analysis was conducted by the University of Alabama at Birmingham (UAB) Animal Physiology Core using an EchoMRI 3-in-1 quantitative magnetic resonance (QMR) machine (Echo Medical Systems, Houston, TX).

### Intraperitoneal Glucose, Oral Glucose, and Insulin Tolerance Test

Mice were fasted 6 h [4 h for insulin tolerance test (ITT)] and blood glucose was measured via tail bleed using a glucose meter (Freestyle Freedom Lite, Abbott Diabetes Care Inc) 0, 15, 30, 60, and 120 min (90 min for ITT) after intraperitoneal glucose (2 g/kg), oral glucose (2 g/kg), or intraperitoneal insulin (1.5 U/kg, Humulin-R) administration.

### Fasting Blood Glucose and Insulin (FBG and FPI)

Mice were fasted for 6 h and blood glucose was determined via tail bleed using a glucose meter (Freestyle Freedom Lite, Abbott Diabetes Care Inc). Briefly, 30–40 µL of blood was collected from the tail using microvette CB300 K2E EDTA dipotassium salt-coated capillary tubes (Sarstedt AG & Co.) and placed on ice. Samples were centrifuged at 1,300 relative centrifugal field (RCF) and the supernatant (plasma) was isolated. Insulin levels were quantified by Rat/Mouse Insulin ELISA kit (Millipore, Cat. No. EZRMI-13K).

### Modified Homeostatic Model Assessment of Insulin Resistance

The HOMA-IR is defined as [fasting blood glucose (FBG) × fasting plasma insulin (FPI)]/22.5, with the constant or normalization factor for human samples derived from the product of the FBG and FPI of a “normal” healthy person (4.5 mmol/L × 5 mU/mL = 22.5) (36). We replaced this constant with the average (FBG × FPI) for control mice, which was 30.5343 mmol/L × 8.3662 mU/mL = 255. Thus, the mHOMA-IR equation used in these studies was (FBG × FPI)/255.

### Oral and Intraperitoneal Glucose Stimulated Insulin Secretion

Mice were fasted for 6 h before collection of 40–50 µL of blood from the tail, and plasma isolated at baseline (0), 5, and 15 min after oral or intraperitoneal administration of glucose (as previously described in FPI methods) to evaluate levels of insulin (Rat/Mouse Insulin ELISA kit, Millipore, Cat. No. EZRMI-13K).

### Leptin Levels

Leptin levels were measured via multiplex immunoassay (Bio-Plex Pro Mouse Diabetes 8-Plex Assay, Cat. No. 171F7001M) performed on plasma isolated from the tail blood of mice fasted for 6 h.

### Collection and Preparation of Tissue for Insulin Signaling

Mice were fasted for 2 h, given a bolus of insulin (1.5 U/kg) or saline followed by euthanasia by pentobarbital administration followed by decapitation 10 min after bolus. Liver and gastrocnemius muscle were collected and immediately frozen in liquid nitrogen followed by storage at −80°C. Samples were homogenized on ice using a motorized homogenizer (Polytron PT 2100, Kinematica AG) in sodium chloride-Tris-EDTA buffer with 0.1% SDS, 0.1% Triton-X, and 1% HALT protease and phosphatase inhibitor cocktail (Thermo Fisher Scientific, Cat. No. 78447). The homogenate was sonicated on ice and centrifuged at 16,000 RCF in a cooled centrifuge at 4°C for 20 min, and the supernatant was collected. An aliquot of each sample was diluted and used to quantify the protein concentration using a Lowry assay. The rest of the sample was aliquoted and frozen in liquid nitrogen and stored at −80°C. Protein samples were diluted to 3 µg/µL concentration in STE buffer with 1% HALT protease and phosphatase inhibitor cocktail for Akt (protein kinase B) immunoblot analyses using primary antibodies total (pan) Akt (Cell Signaling, Cat. No. 4691) and phosphorylated Akt (p-Akt) Ser473 (Cell Signaling, Cat. No. 4060) and anti-rabbit IgG HRP-linked secondary antibody (Cell Signaling, Cat. No. 7074) to assess for changes in insulin signaling.

### Statistical Analyses

Results are expressed as means ± SE. ANOVA was used to test the null hypothesis that all samples were drawn from a single population and if the significant differences existed (*P* < 0.05), a Student–Newman–Keuls test was used for group comparisons. All analyses were carried out using SigmaStat statistical software.

## RESULTS

**Figure 1.**
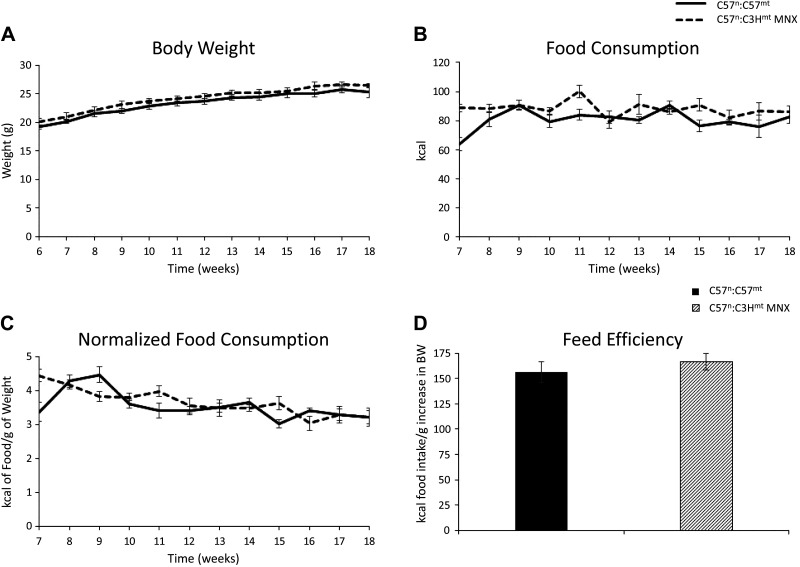
Body weight and food consumption. *A*–*C*: solid and dashed lines indicate C57^n^:C57^mt^ and C57^n^:C3H^mt^ mice, respectively. *A*: average weekly body weight of singly housed C57^n^:C57^mt^ and C57^n^:C3H^mt^ mice (control and MNX in text, respectively), commencing at 6 wk until 18 wk of age. *B*: average weekly food consumption per mouse. *C*: food consumption normalized to body weight. *D*: total food intake normalized to total increase in body weight from *weeks 6* to *18* (feed efficiency). No differences were observed (*n* = 6–9 animals per group). BW, body weight; MNX, mitochondrial-nuclear eXchange.

**Figure 2.**
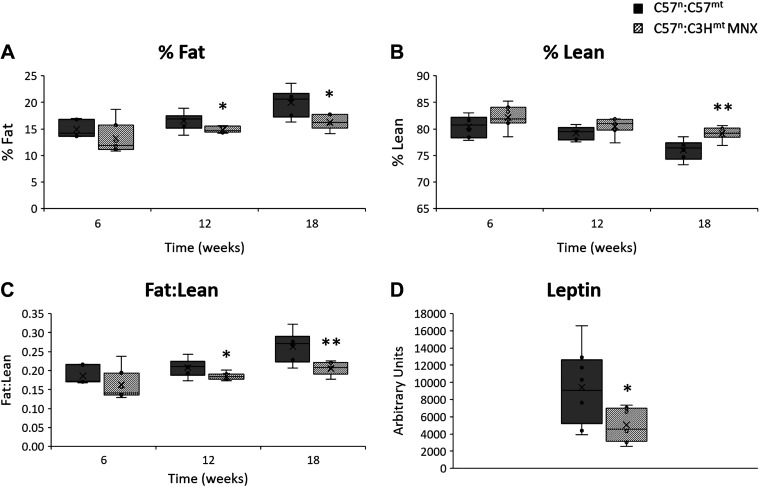
Body composition and leptin levels. *A*–*C*: QMR was used to assess body composition changes in mice at 6, 12, and 18 wk of age. *A*: percent fat normalized to body mass [% = fat (g)/body mass (g)]. *B*: percent lean normalized to body mass [% = lean (g)/body mass (g)]. *C*: ratio of fat to lean mass [fat (g)/lean (g)]. C57^n^:C57^mt^ WT mice. *D*: mean plasma leptin levels in 18-wk-old C57^n^:C57^mt^ and C57^n^:C3H^mt^ MNX mice. Asterisk(s) indicate a significant difference exists between indicated groups, *P < 0.05, **P < 0.01 (*n* = 6–10 animals per group). MNX, mitochondrial-nuclear eXchange; QMR, quantitative magnetic resonance; WT, wild type.

No differences in weight (Fig. 1*A*) or food consumption (Fig. 1*B*) were observed between control and corresponding MNX strains. Normalization of caloric intake by weight also showed no differences between groups (Fig. 1*C*), indicating that food consumption was consistent with body weight and that feed efficiency was similar between groups (Fig. 1*D*). However, MNX mice had significantly decreased adiposity (% fat) relative to control mice by 12 wk of age, which was maintained at 18 wk (Fig. 2*A*). Consistently, lean mass (%) was significantly increased at 18 wk of age in the MNX mice relative to control animals (Fig. 2*B*); correspondingly, the fat to lean ratio was significantly decreased in these mice relative to the controls by 12 and 18 wk of age (Fig. 2*C*). Consistent with its leaner phenotype, MNX mice exhibited reduced circulating leptin levels at 18 wk of age as compared with control mice (Fig. 2*D*).

**Figure 3.**
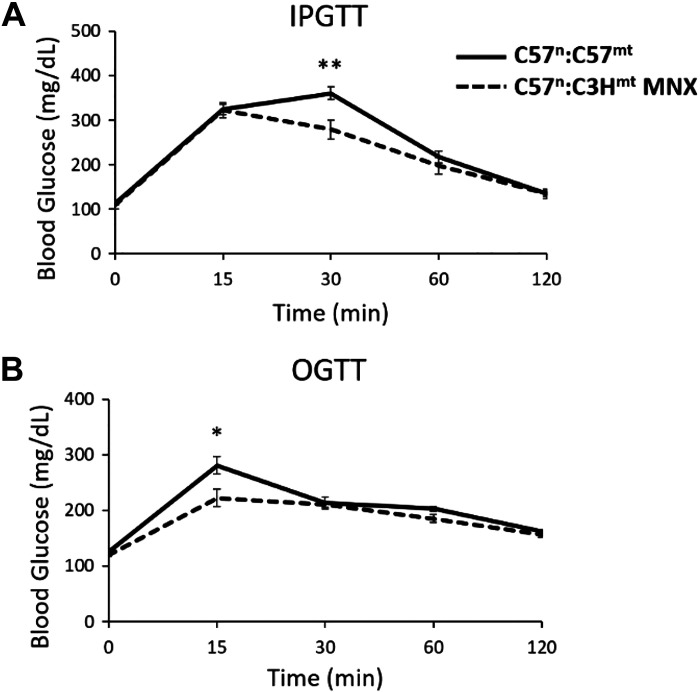
Eighteen-week intraperitoneal and oral glucose tolerance tests. Mice were fasted for 6 h and given 2 g/kg glucose intraperitoneally (*A*) or orally (*B*), and blood glucose measured at 0, 15, 30, 60, and 120 min after glucose administration. Solid and dashed lines indicate C57^n^:C57^mt^ (control in text) and C57^n^:C3H^mt^ (MNX in text) mice, respectively. Asterisk(s) indicate a significant difference exists between groups, *P < 0.05, **P < 0.01 (*n* = 7–9 animals per group). IPGTT, intraperitoneal; MNX, mitochondrial-nuclear eXchange; OGTT, oral glucose tolerance tests.

**Figure 4.**
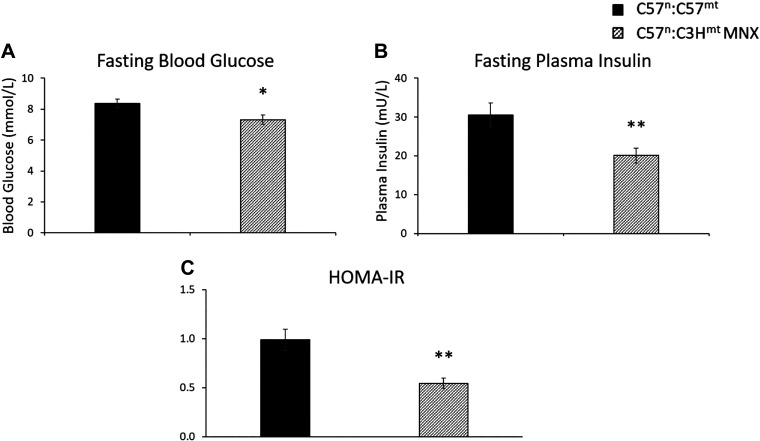
Fasting blood glucose, fasting plasma insulin, and modified Homeostatic Model Assessment of Insulin Resistance (mHOMA-IR). Mice were fasted for 6 h and fasting blood glucose (*A*) and fasting plasma insulin (*B*) levels determined, which were used to calculate mHOMA-IR (*C*: see materials and methods). Asterisk(s) indicate a significant difference exists between C57^n^:C57^mt^ control and C57^n^:C3H^mt^ MNX groups, *P < 0.05, **P < 0.01 (*n* = 9 animals per group).

To interrogate glucose homeostasis, both intraperitoneal (IPGTT) and oral (OGTT) glucose tolerance tests were performed in 18-wk-old animals (Fig. 3). Consistent with a leaner phenotype (Fig. 2), MNX mice appeared more glucose tolerant than their nuclear matched controls, employing either IPGTT or OGTT approaches (Fig. 3, *A* and *B*, respectively). Similarly, MNX mice had significantly lower fasting blood glucose (FBG, Fig. 4*A*) and fasting plasma insulin (FPI, Fig. 4*B*) levels, contributing to a lower mHOMA-IR (Fig. 4*C*) compared with their nuclear-matched control counterparts, suggesting increased insulin sensitivity in the MNX animals.

**Figure 5.**
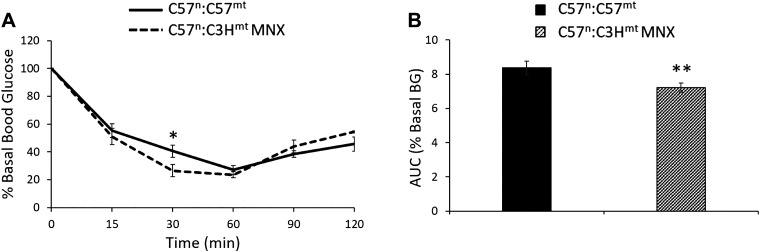
Intraperitoneal insulin tolerance tests in 18-wk-old mice. Mice were fasted for 4 h and blood glucose levels (*A*) measured at 0, 15, 30, 60, 90, and 120 min after insulin (1.5 U/kg ip) administration. *B*: area under the curve from 0 to 30 min. Asterisk(s) indicated a significant difference exists between groups, *P < 0.05 and **P < 0.01 (*n* = 7–9 animals per group). AUC, area under the curve; BG, blood glucose; MNX, mitochondrial-nuclear eXchange.

To test the hypothesis that the MNX mice harboring the C3H mtDNA would be more insulin sensitive than control mice, intraperitoneal insulin tolerance tests (IPITT) were performed on both strains of mice. The IPITT results were consistent with the mHOMA-IR, confirming that the MNX had increased insulin sensitivity relative to the control mice (Fig. 5). While performing these and other studies, we noted that 43.5% (10/23) of the MNX mice compared with 6.7% (2/30) of their control counterparts experienced exogenous insulin-induced hypoglycemic shock (animals experiencing hypoglycemic shock were administered a glucose rescue bolus after the 30’ timepoint; hence, data from these animals are only included up to the 30 min timepoint), requiring a intraperitoneal bolus of glucose for rescue (*P* = 0.004), consistent with increased insulin sensitivity in MNX mice harboring the C3H mtDNA relative to control counterparts.

**Figure 6.**
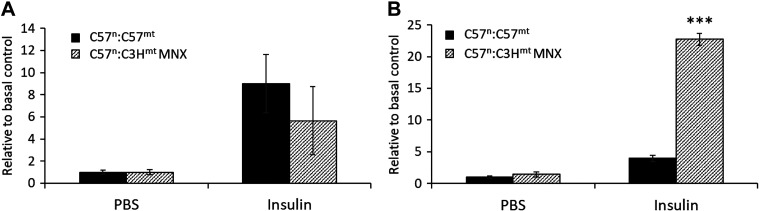
Phosphorylated to total Akt ratios in liver and gastrocnemius muscle. Mice were fasted for 2 h and given either PBS or insulin (1.5 U/kg) intraperitoneally. Tissues were harvested after 10 min and snap-frozen in liquid nitrogen, and tissue homogenates were used for subsequent Western blotting (see materials and methods) to determine levels of phosphorylated Akt/total Akt in liver (*A*) and gastrocnemius muscle (*B*). Levels are expressed relative to basal (PBS treated) levels observed in C57^n^:C57^mt^ control group. Asterisks indicate a significant difference was observed between indicated groups, ***P < 0.001 (*n* = 5–6 animals per group). MNX, mitochondrial-nuclear eXchange.

Insulin can trigger a cascade of signaling events that include phosphorylation of Akt (p-Akt), which promotes glucose uptake by the muscle and adipose tissues, while inhibiting gluconeogenesis by the liver. Consequently, p-Akt to total Akt (t-Akt) levels were assessed in liver and muscle tissues to determine p-Akt/t-Akt ratios from control and MNX liver and gastrocnemius muscle tissues (Fig. 6 and Supplemental Fig. S1; see https://doi.org/10.6084/m9.figshare.15035775). In liver tissue, no differences were observed in p-Akt/t-Akt ratios (Fig. 6*A*); however, insulin-stimulated Akt phosphorylation was elevated in gastrocnemius muscle of the MNX mice harboring the C3H mtDNA as compared with control mice (Fig. 6*B*), consistent with the noted increased glucose tolerance, lower fasting blood glucose, and fasting plasma insulin levels in MNX mice compared with controls (Figs. 4 and 5).

## DISCUSSION

Glucose metabolism is a critical factor for growth and survival. To this end, multiple endocrine signals, including insulin and glucagon (secreted by pancreatic β and α cells of islets of Langerhans, respectively), maintain glucose homeostasis. Mitochondria play an integral role in normal glucose metabolism and homeostasis, in that, glucose is converted to reducing equivalents that can be utilized by the organelle to generate ATP, for a variety of cellular functions—including insulin delivery to the circulation. In this respect, we were interested in determining whether different mtDNAs could influence aspects of glucose metabolism. Prior studies in humans have described associations of different mtDNA mutations with elevated fasting insulin, glucose, and HOMA-IR values (4–6), as well as associations with type 2 diabetes (7, 8) and maternally inherited forms of diabetes (1, 2, 37). Consequently, circumstantial evidence derived from human studies clearly suggests an association between differences in glucose metabolism and mtDNA haplotype.

For these studies, we were interested in directly defining the impact of mtDNA haplotype on “normal” glucose metabolism. We hypothesized that nonpathogenic mtDNAs from a mouse less susceptible to metabolic diseases (e.g., C3H/HeN) could improve aspects of glucose metabolism, including glucose tolerance and insulin sensitivity, when placed on the same nuclear genetic background as a more susceptible mouse strain (C57BL/6J). To directly address this question, we utilized the MNX mouse model to generate animals that were isogenic in terms of their nuclear background (C57BL6/J) but would have distinct mtDNAs (20–23).

Results from these studies showed that mtDNA background affected body composition, glucose tolerance, and insulin sensitivity. mtDNA background did not appear to significantly impact body weight, food intake, or feed efficiency in these studies within the observed experimental design, despite the linkage of the C3H/HeN mtDNA in MNX mice with a leaner phenotype by 12 wk that persisted at 18 wk of age, indicative of differences in caloric utilization and biomass storage. By 18 wk of age, MNX mice were more glucose tolerant than control mice, regardless of route of glucose administration (IPGTT or OGTT) and were more insulin sensitive as measured by mHOMA-IR (lower FBG and FPI levels), response to exogenously administered insulin (IPITT), and muscle Akt phosphorylation as compared with their control counterparts. Interestingly, we observed no difference in liver insulin-stimulated Akt phosphorylation, suggesting that these effects may be sequestered to skeletal muscle. Although insulin-stimulated Akt phosphorylation was not measured in adipose tissue, it is possible that differences in adipose insulin sensitivity may also contribute to overall insulin sensitivity in these mice. Akt phosphorylation is an important step of insulin signaling as it stimulates GLUT4 translocation in muscle and adipose and glycogen synthesis in the liver, both of which increase glucose uptake by these tissues. However, there are many upstream proteins in this pathway that also can be measured to evaluate insulin sensitivity in future studies, including the insulin receptor (IR), which autophosphorylates (pIR) on insulin binding and in turn phosphorylates the insulin receptor substrate (pIRS). In addition, Akt phosphorylation at the Thr308 site can also be measured in addition to the Ser473 site as done in this study, along with a host of other intermediate kinases and proteins.

We have previously shown that different mtDNA backgrounds on the same nuclear genetic background can significantly alter whole animal metabolic efficiency and body composition in mice, without affecting activity or food consumption (23). Similarly, we have shown that these same mtDNA-nDNA combinations significantly alter aspects of electron transport and metabolic efficiency, or “mitochondrial economy.” Mitochondrial economy refers to the utilization of electron energy for proton pumping that drives mitochondrial membrane potential, which is utilized by ATP synthase for the production of ATP. Fetterman and coworkers reported that C57BL/6J mice having the C57 mtDNA (C57^n^:C57^mt^) had significantly higher levels of mitochondrial economy relative to C57BL/6J mice harboring the C3H/HeN mtDNA (C57^n^:C3H^mt^), and this difference was due to a guanosine to adenosine transition at nucleotide 9348 in subunit III of cytochrome c oxidase, resulting in highly conserved valine being replaced by isoleucine at AA248. Fetterman and colleagues showed that this cytochrome c oxidase mutation caused a decrease in the activity of this enzyme in mice with C3H/HeN mtDNA as compared with mice with C57BL/6J mtDNA (21). In this case, our findings that the control (C57^n^:C57^mt^) mice have greater adiposity compared with the MNX (C57^n^:C3H^mt^) are consistent with the finding of differences in mitochondrial economy between these two groups as previously reported (24). A question that arises is whether the observed differences in body composition were causally related to differences in glucose metabolism between the MNX and control mice—while potentially a complex endeavor due to the fact MNX and control mice respond differently to the same diet (herein and Ref. 23), studies of pair fed mice designed to achieve similar/same body compositions would potentially shed greater light on this question.

In this study, we also assessed circulating leptin levels. Leptin, the prototypical adipokine, circulates at levels directly proportional to body fat (38, 39) and acts on neurons of the hypothalamic arcuate nucleus to decrease food intake and increase energy expenditure (39, 40). Elevated leptin levels are positively associated with increased type 2 diabetes risk probably due to the development of leptin resistance (40). Consistent with the body composition results in this study, MNX mice had decreased leptin levels compared with control animals; thus, it would be interesting to determine if the observed differences in body composition between the control and MNX mice are the result of leptin levels and/or overall changes in body metabolism. Previous studies using the C57BL6/J strain showed that mtDNA background had a differential impact on transcriptional response in adipose tissue to a dietary challenge (high fat diet), with mice carrying the C57BL/6J mtDNA having an approximately twofold greater response (in number of differentially expressed genes) relative to those harboring the C3H/HeN mtDNA (23). Consistent with the observations herein, genes associated with metabolic processes were those with the highest levels of differential expression in that study, followed with biological regulation and response to cellular stimuli (23).

Two nonsynonymous mutations in protein coding subunits exist between the C57BL6/J and C3H/HeN mtDNAs (subunit 3 in complex I and subunit 3 in complex IV) (41). The C57BL/6J mtDNA is linked to greater mitochondrial economy (electron flow utilized for making equivalent amounts of ATP) and oxidant production relative to the C3H/HeN mtDNA (21). Because mitochondria are sources of ATP, metabolites, heat, and oxidants, features of these basic functions also enable it to participate in redox signaling, immune response, and glucose homeostasis (42–50). The electron transport chain (ETC) and citric acid cycle (CAC) are directly intertwined and at the nexus of these processes, with the former supplying energy (molecular and thermal) and oxidants and latter providing metabolic intermediates for various cellular pathways. Recently, it has been noted that changes in levels of mitochondrial metabolic intermediates (via the CAC) also impact histone acetylation and methylation, which directly links changes in mitochondrial metabolism with gene expression (51, 52). Although studies have suggested that mutations in the nuclear encoded nicotinamide nucleotide transhydrogenase (*Nnt*) gene in the C57BL/6J relative to the C57BL/6N are associated with changes in insulin secretion and impaired glucose tolerance in the C57BL/6J mouse (30, 53), others have questioned differences that appear to exist when performing in vitro versus in vivo studies (54); however, both the C57 control and MNX (harboring the C3H mtDNA) mice in this study shared the same nuclear genetic background. Hence, the observed differences herein are likely attributable to the different mtDNA backgrounds on the same nuclear genome.

In sum, these studies provide direct evidence that the mtDNA background can impact normal glucose metabolism in the C57BL/6J mouse and demonstrate that different mtDNA combinations on the same nuclear background can alter body composition toward a leaner phenotype. Consequently, the results of this study are consistent with the concept that nonpathogenic mtDNA polymorphisms, which change aspects of organelle metabolism, even modestly, can have a sustained effect on multiple cellular pathways by virtue of the mitochondrion’s central and systemic role in metabolism.

## SUPPLEMENTAL DATA

Supplemental Fig. S1: https://doi.org/10.6084/m9.figshare.15035775.

## GRANTS

This work was supported by National Institutes of Health Research Award (1RO1 HL103859 to S.W.B and 5R01DK112934 to K.M.H) and Training Program in Cardiovascular Pathophysiology (T32HL007918 to S.W.B. and M.J.S.).

## DISCLOSURES

No conflicts of interest, financial or otherwise, are declared by the authors.

## AUTHOR CONTRIBUTIONS

M.J.S., K.M.H., and S.W.B. conceived and designed research; M.J.S., J.A.B., and C.H. performed experiments; M.J.S., J.A.B., K.M.H., and S.W.B. analyzed data; M.J.S., A.W.C., K.M.H., and S.W.B. interpreted results of experiments; M.J.S. prepared figures; M.J.S. drafted manuscript; M.J.S., A.W.C., J.A.B., C.H., K.M.H., and S.W.B. edited and revised manuscript; M.J.S., A.W.C., J.A.B., C.H., K.M.H., and S.W.B. approved final version of manuscript.
